# Cultural adaptation and reliability of the General Comfort Questionnaire for chronic renal patients in Brazil

**DOI:** 10.1590/1518-8345.2280.2963

**Published:** 2017-12-21

**Authors:** Geórgia Alcântara Alencar Melo, Renan Alves Silva, Francisco Gilberto Fernandes Pereira, Joselany Áfio Caetano

**Affiliations:** 1 MSc, Nursing, MSc in Enfermagem pela Universidade Federal do Ceará, PhD, PhD in Nursing, Universidade Federal do Ceará, Fortaleza, Ceará, Brazil; 2 MSc, Nursing, MSc in Nursing at Universidade Federal do Ceará, Fortaleza, Ceará, Brazil.; 3 MSc, Nursing, MSc in Nursing at Universidade Federal do Ceará, Adjunct Professor, Nursing School, Universidade Federal do Piauí, Picos, PI, Brazil.; 4 PhD, Nursing, PhD in Nursing at Universidade Federal do Ceará, Associated Professor, Nursing School, Universidade Federal do Ceará, Fortaleza, CE, Brazil

**Keywords:** Translations, Validation Studies, Renal Insufficiency, Renal Dialysis, Nursing, Nursing Theory

## Abstract

**Objective:**

to make the cultural adaptation and evaluate the reliability of the Brazilian version of the *General Comfort Questionnaire* for chronic hemodialytic renal patients.

**Method:**

methodological study with the following steps: translation; consensus among judges; back-translation; validation of equivalence (semantic, idiomatic, experimental and conceptual) by 12 judges; and pre-test with 80 chronic renal patients on hemodialysis. Reliability was checked through measures of internal consistency (*Cronbach*’s alpha).

**Results:**

the overall consensus of the instrument had 94.3% of equivalence. Twenty-one items of the instrument were modified. Of these, only two needed semantic and idiomatic changes. The other 19 underwent few modifications, such as reversing words in the sentence and replacing some corresponding synonym terms. The *Cronbach*’s alpha was 0.80, indicating optimal internal consistency. In the application, the total score ranged from 116 to 172 points (M = 151.66; SD = ± 12.60).

**Conclusion:**

the validation of the Portuguese version of the instrument represents one additional resource to be made available to nephrologist nurses; it will aid in directing the decision-making so that the nursing interventions be performed according to the level of comfort and domain, either physical, socio-cultural, environmental or psycho-spiritual. The tool was named in Portuguese: General Comfort Questionnaire - Brazilian version.

## Introduction

The need for comfort is common to all people at any stage of their life cycle. However, in situations when health is compromised, in the presence of a disease, in socially vulnerable situations or others that leave people more vulnerable, this concept gains particular interest, given its scope and applicability.

Comfort is an individual and subjective concept, comprising physical, environmental, social and psycho-spiritual aspects. It is a dimension of holistic care and can occur to a greater or lesser extent depending on different factors of individuals and their personal perceptions. Thus, physical symptoms, environmental organization, interpersonal relationships, beliefs, and individual values ​​are related to the patients’ experiences during care. 

Comfort is defined as the state in which the need for relief, tranquility, and transcendence are strengthened in the four contexts of human experience: physical, psycho-spiritual, sociocultural, and environmental, and means the result of help, the subjective experience of the momentary state in which the person perceives herself calm, relieved or able to overcome the discomfort.

On the basis of this definition, Kolcaba built the *General Comfort Questionnaire* (GCQ), an instrument to measure comfort and identify positive and negative aspects in providing care to a patient regardless of his/her health condition. In this sense, instruments capable of measuring and stratifying comfort levels of patients in the clinical practice is essential to evaluate the comfort resulting from nursing actions.

The *GCQ* is a generic, self-report and strongly recommended instrument for descriptive and interventional studies. It has 48 items covering the physical, spiritual, environmental and social dimension. 

The application of the comfort questionnaire runs through several clinical contexts, as has been adapted to more specific areas, such as: pediatrics; psychiatry; lesbian, gay, bisexual and transvestite (LGBT) populations; deaf patients; immobilized patients; childbirth and postpartum; perianesthesia; urinary incontinence; radiotherapy; end-of-life planning (for patients and families); touch of healing; nurses; caregivers; patients with breast cancer; caregivers of women with terminal cancer; and even for architecture, in which the researcher reflects on the idea of ​​comfort from the built environment. 

Notably, the general comfort questionnaire has already been translated and validated for different languages ​​and sociocultural contexts, such as Spanish, Italian and Turkish. In the Portuguese language, there are versions of the instrument aimed at psychiatric and breast cancer patients, translated and validated into Portuguese from Portugal. 

As for the Brazilian Portuguese, the translations are: *End of Life Comfort Questionnaire* and *Holistic Comfort Questionnaire - caregiver.* The final version of the *End of Life Comfort Questionnaire* has been applied to patients with heart failure under outpatient care; and the instrument that evaluates the overall well-being of caregivers have been widely applied to caregivers of people with cancer. 

By fostering comfort, nurses help patients to effectively overcome the moment of transition experienced. Here we focus attention on chronic renal patients in hemodialysis therapy. Renal disease impacts the personal and professional life of individuals, and causes complex therapeutic regimens and significant necessary changes in activities of daily living. 

The clinic where the patients receive treatment should also be considered in the context of comfort, because patients stay on average four hours in three days a week there, undergoing treatment in armchairs, by means of fistulas or catheters.

Since usability in different contexts directs nursing interventions and assists in decision making based on the identification of comforting factors, the application of the general comfort questionnaire in the population of chronic renal patients undergoing hemodialysis may contribute to increase their well-being as well as direct health promotion actions. 

In view of the foregoing, the present study aims to make the cultural adaptation and evaluate the reliability of the Brazilian version of the *General Comfort Questionnaire* for chronic renal patients in hemodialysis therapy.

## Method

Methodological study, quantitative, with cross-sectional design. The cultural adaptation and validation of the *GCQ*, used to measure the comfort level of patients based on self-perception, was carried out.

The process was carried out in accordance with international standards for adaptation of measuring instruments^10^, and involved six sequential steps: initial translation; synthesis of translations; back-translation to the source language; review by a committee of judges; pre-test; and, final review of the adaptation by the researchers. This stage occurred between May and September 2016.

Initial translation: The first stage, or initial translation, was carried out independently by two certified public translators, one with background in health (“clinical” translator - T1) and the other, a lay translator regarding the health area (“blind” translator - T2). 

Synthesis of translations: a meeting was held with the purpose of synthesizing the two versions. A committee composed of four judges was formed, including: two certified public translators with comprehensive knowledge of English; a researcher of this methodology; and one of the authors of the present study. Semantic, cultural and idiomatic equivalences were examined at this moment. At the end, a consensual version of the instrument was obtained (T12).

Back-translation: the unified version (T12) was translated into English by two US citizens living in Brazil. The two versions of this step were also harmonized into a final single back-translated version (BT12), which was grammatically and semantically equivalent to the original questionnaire.

Committee review: a committee of judges was selected for this step, with the function of preparing a pre-final Portuguese version of the instrument. Among the inclusion criteria for judges were: fluency in the English language and experience in translation and validation of research instruments. The instruments used in this step were organized in a folder and delivered to the 12 selected judges. The following documents were provided: (1) an invitation letter and the informed consent form; (2) a form with items addressing biographical data; (3) all versions of the questionnaire (original, T1, T2, T12, BT1, BT2 and BT12); (4) a form to evaluate the scale items. All versions were carefully examined by a committee of 12 judges for semantic, idiomatic, experimental, and conceptual aspects. A period of 15 days was set for the judges to review and return the completed instruments. Changes in expressions or similar words for the pre-final version were suggested at this stage. 

It is worth emphasizing that semantic equivalence considers the adaptation of words according to the grammar and vocabulary of the translated language; idiomatic equivalence refers to informal colloquial expressions, or slang used in the country of origin; the experimental equivalence refers to identifying whether the adapted version expresses the experience as in the daily life of the Brazilian cultural context; the conceptual equivalence consists in verifying the words that have conceptual connotation are adequate to the context of Brazilian health services.^11^

Each judge evaluated the 48 questions in these four aspects, totaling 192 items. The consensus between the judges of ≥ 80% was established for each item evaluated, and those that did not obtain a consensus were discussed in a face-to-face meeting between the investigators and the individual judges, at the moment of returning the folders. On this occasion, decisions were made regarding the pre-finalization of the translated instrument, including all its components: items, instructions and response format.

Pre-test: A pre-test was applied in order to confirm if the questionnaire had adequately comprehensible items for the evaluation of comfort level. This was done with 80 chronic renal patients in hemodialysis therapy in a private clinic of renal replacement therapy in the city of Patos-PB. Inclusion criteria were: to be undergoing dialysis for at least six months, to be older than 18 years, to achieve a Glasgow scale score of 15, and to present clinical conditions and cognitive ability to respond to the instrument.

Chronic renal patients in hemodialysis therapy were chosen because they experience changes in their daily life imposed by their becoming dependent on treatment that requires periodicity, and also learn to live with symptoms considered uncomfortable, such as nausea, vomiting, hypotension and fatigue.^12^ ^-^ ^13^

The period of application of the pre-test was November to December 2016, and all the approaches for participation in the research were done individually and privately. Patients answered the questionnaire as they were in dialysis.

Final review of the adaptation by the researchers: after the application of the pre-test, there was a meeting between the researchers in order to verify whether there had been any difficulty of understanding by the target public, so that the appropriate adjustments could be made. 

The *Cronbach’s α coefficient* was used to measure the reliability or internal consistency of the instrument. The ideal range of alpha values ​​is considered to be between 0.7 and 0.9.^14^

Prior to the methodological steps of this study, the formal authorization of the researcher responsible for the creation of the instrument was obtained by electronic mail. The study respected all the ethical aspects of national and international research involving human beings, with approval nº 1.482.596.

## Results

Before the process of validation of equivalences carried out by the judges, a general equivalence of 94.3% was obtained. Regarding the validity of the instrument, the reviewing committee indicated a consensus of 100% of concordance in 27 items of the instrument, evaluating them as pertinent and ensuring their semantic, cultural, idiomatic and conceptual coherence, which did not undergo any changes. 

**Figure 1:**
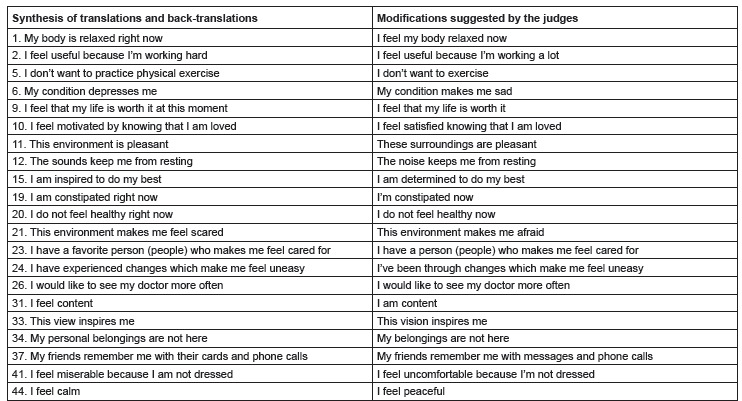
Systhesis version of modified items after submission to content validity by judges. Fortaleza; Ceará; Brazil, 2016

Twenty-one items of the instrument were modified. Of these, only two (items 37 and 41) underwent semantic and idiomatic changes (Figure 1). The other 19 items underwent few modifications. Among the changes made are: changes in the grammatical order, with inversion of words in the sentence; and replacement of some corresponding synonym term or deletion of words. 

In the question 1, “My body is relaxed right now”, the judges suggested rephrasing the item as “I feel my body relaxed now”. In the question 2, “I feel useful because I am working hard”, there were suggestions to modify the term hard, and use a lot, since, a lot it refers to heavy work, with serious physical discomforts. 

In the question 5, “I do not want to practice physical exercise”, the judges suggested rephrasing the term practice by simply exercise because the word practice would make the understanding difficult in the case of patients with lower instructional level. In the question 6, “My condition depresses me”, the term “depresses me” was suggested to be replaced by “makes me sad,” since not everyone knows the term “depressed”.

In the question 10, “I feel motivated by knowing that I am loved” was suggested to be modified to “I feel satisfied knowing that I am loved”, because the term motivated is far from reality of the study population. 

In the question 12, “The sounds keep me from resting” was suggested to be rephrased as “The noise keeps me from resting”, since two judges justified that the expression used is considered abstract and generalist, taking into consideration that some people initiate and maintain a pattern of sleep while listening to instrumental songs (sounds) capable of favoring muscle relaxation. With this, it was decided to change the term to “noise”, as this is more suitable to the idea conveyed by the item. 

In the question 15, “I am inspired to do my best”, it was suggested that the term “inspired” be replaced by “determined”; and in the question 21, “This environment makes me feel scared” was suggested to be modified to “This environment makes me afraid”. The changes were justified by the better known words. 

In the question 23, “I have a favorite person (people) who makes me feel cared for”, it was suggested to be replaced by “I have a person (people) who makes me feel cared for”, because the term favorite is not relevant. In the question 33, “This view inspires me” was suggested to be modified to “This vision inspires me”. 

In the question 37, “My friends remember me with their cards and phone calls” was suggested to change to “My friends remember me with messages and phone calls”. The judges evaluated the item considering the cultural context of the country, and in the Brazilian reality, people do not have the habit of sending cards to patients while hospitalized. In this sense, they agreed that the term could be replaced by telephone messages. 

In the question 41, “I feel miserable because I am not dressed” was suggested to be changed to “I feel uncomfortable because I’m not dressed”. In this question, the judges justified that the term miserable could hinder the understanding of the participants of the study, because, despite being a colloquial term, the Canadian reality is incompatible with the sociocultural context and life experiences of chronic kidney patients. Other terms were deemed similar to the Brazilian reality, not compromising the investigation, such as terrible, unpleasant, bad and upset. In the question 44, “I feel calm” was suggested to be changed to “I feel peaceful”.

A small amount of patients (5%) reported difficulty answering an item, with respect to terms used, being necessary that researchers explained the item. Only after an explanation of the term, the question was understood by the patients. Thus, the terms were replaced by others for better understanding, such as “I am inspired to do my best” by “I am determined to do my best”.

The pre-test was applied to 80 chronic renal patients on hemodialysis. The majority were male (56.3%), black and/or brown skinned (67.5%), with a partner (61.3%), with average schooling up to six years (62.5%), retired (72.5%), with income of one minimum wage (87.5%), Catholic (90.0%), and with a poor perception of their own health (65.0%). 

The age ranged from 19 to 89 years, with a mean of 54.32 (± 17.16) years. Schooling ranged from zero to 20 years, with a mean of 6.21 (± 4.89) years. Regarding clinical data, patients with systemic arterial hypertension (36%), use of arteriovenous fistula (75%) and undergoing dialysis treatment for up to three years (65%) predominated. The mean time of dialysis was 3.94 years, with minimum and maximum time of eight months and 20 years, respectively.

Regarding the application of the QCG - Brazilian version, interviews were preferred instead of self-report, and the time needed to fill the instrument ranged from 14 to 35 minutes (M = 25.6 min.; SD = ±4.24). There was a high percentage of patients with low schooling (< 6 years) in the population studied. In the elderly, the time ranged from 19 to 45 minutes (M = 32.4 min.; SD = ± 5.32). Participants were unanimous in considering the questionnaire easy to understand. 

The total score ranged from 116 to 172 points (M = 151.66; SD = ± 12.60). Men obtained an average of 153.48 (± 12.45) while women, 149.94 (± 12.58). Regarding the age group, patients aged 19 to 54 years averaged 154.11 (± 11.66), and patients aged 55 or more years, 148.94 (± 13.19). 

The *Cronbach’s* alpha was 0.80, indicating optimal internal consistency. The item-total correlation revealed that all items had an item to item *Cronbach’s* alpha greater than 0.70. With this, we decided to keep all items of the instrument for further analysis. It was observed that the exclusion of the item 43 “I am alone, but not lonely” led to a *Cronbach’s* alpha of 0.818, and the variance increased to 226.90 (Table 1). However, in view of the importance of the item for comfort assessment, we decided to keep it.

**Table 1:** Internal consistency coefficient of *Cronbach’s* alpha of the General Comfort Questionnaire (QCG) - Brazilian version. Fortaleza, CE, Brazil, 2016

QCG items*	**Average if item is** **excluded**	**Variance if item is excluded**	**Correlation item/** **Total corrected**	*Cronbach’s alpha* **if the item is excluded**
**43**	**149.4375**	**226.907**	**-0.283**	**0.818**
**16**	**149.0750**	**218.906**	**-0.066**	**0.813**
**25**	**148.1875**	**217.167**	**-0.011**	**0.809**
**33**	**149.3000**	**218.086**	**-0.036**	**0.809**
**5**	**148.9750**	**213.088**	**0.073**	**0.808**
**10**	**147.7625**	**218.918**	**-0.072**	**0.805**
**15**	**148.2625**	**216.854**	**0.023**	**0.805**
**4**	**150.4625**	**218.682**	**-0.130**	**0.804**
**22**	**148.1000**	**214.648**	**0.094**	**0.804**
**27**	**147.8625**	**214.626**	**0.100**	**0.804**
**12**	**148.1750**	**211.969**	**0.152**	**0.803**
**37**	**147.8625**	**214.930**	**0.102**	**0.803**
**38**	**147.5500**	**216.884**	**0.161**	**0.802**
**2**	**148.0000**	**212.456**	**0.192**	**0.801**
**11**	**147.8000**	**213.377**	**0.199**	**0.801**
**23**	**147.5750**	**214.602**	**0.265**	**0.801**
**44**	**148.0000**	**212.253**	**0.188**	**0.801**
**8**	**149.2750**	**207.366**	**0.252**	**0.800**
**13**	**148.5750**	**208.703**	**0.246**	**0.800**
**17**	**147.8250**	**211.437**	**0.228**	**0.800**
**21**	**148.0875**	**209.929**	**0.236**	**0.800**
**36**	**148.4125**	**208.397**	**0.239**	**0.800**
**6**	**148.9125**	**206.511**	**0.270**	**0.799**
**47**	**147.7250**	**211.797**	**0.327**	**0.799**
**3**	**148.4500**	**209.339**	**0.297**	**0.798**
**19**	**147.8375**	**210.391**	**0.330**	**0.798**
**9**	**147.8500**	**209.648**	**0.354**	**0.797**
**26**	**149.0000**	**204.962**	**0.326**	**0.797**
**32**	**147.8750**	**208.187**	**0.347**	**0.797**
**40**	**148.2125**	**206.347**	**0.325**	**0.797**
**42**	**147.9750**	**208.354**	**0.326**	**0.797**
**45**	**148.1125**	**206.987**	**0.323**	**0.797**
**46**	**148.0625**	**209.021**	**0.387**	**0.797**
**31**	**147.7250**	**208.632**	**0.445**	**0.796**
**34**	**148.4125**	**204.828**	**0.353**	**0.796**
**41**	**147.7250**	**209.417**	**0.462**	**0.796**
**7**	**147.8625**	**207.538**	**0.461**	**0.795**
**29**	**148.2000**	**205.504**	**0.419**	**0.795**
**30**	**147.9500**	**207.187**	**0.413**	**0.795**
**39**	**149.3875**	**204.291**	**0.374**	**0.795**
**14**	**149.0750**	**202.096**	**0.419**	**0.794**
**18**	**147.9875**	**204.620**	**0.433**	**0.794**
**35**	**148.2875**	**203.676**	**0.440**	**0.794**
**48**	**149.7750**	**202.658**	**0.427**	**0.794**
**1**	**148.2500**	**201.962**	**0.499**	**0.792**
**20**	**148.5625**	**199.388**	**0.464**	**0.792**
**28**	**147.9375**	**204.135**	**0.522**	**0.792**
**24**	**148.2000**	**201.605**	**0.519**	**0.791**

*General Comfort Questionnaire

## Discussion

Comfort, or comfort care, has gained greater visibility in palliative care literature, and its goal is to provide immediate relief through a set of basic interventions^15^. Thus, it is necessary that besides performing such interventions, professionals be able to evaluate their effectiveness from the point of view of the patients themselves and also of their families, from which comes the justification of the need to use consistent instruments. 

The *CGQ,* addressed in the present study to meet this demand, was analyzed by the judges and patients who participated in its adaptation to Brazil, respectively, as a useful and relevant instrument for daily practice in the clinical context. Most participants said that the items were easy to understand, as to their level of understanding.

In the current scenario of clinical practice, studies that help understand the patients’ perceptions and attitudes related to general comfort and their levels are fundamental. They make it possible to detect the modifiable factors capable of improving the physical, psychological, environmental and spiritual well-being.^16^

The process of cross-cultural adaptation of the questionnaire required grammatical and semantic adjustments, considering the cultural context and the use in clinical practice. Only one item was modified semantically (item 37), because with the current technological advance, saying that friends only remember with cards and phone calls can be considered a bias for research, as one of the main forms of communication nowadays are the social networks such as *Facebook* and *Whatsapp.* Thus, this item was modified to “My friends remember me with messages and phone calls”.

The internet has changed the way people work and the relationship between people and with the world. The messages are now digital, arrive in seconds, by clicking a key, at the touch of fingers or by voice command. 

Virtually created social support networks are considered strong tools for increasing adherence to theraphy and improving knowledge about aspects of the health-disease process by patients and their families. Moreover, they favor the possibility of interactions, somehow decreasing communication isolation, what may improve the sense of comfort and well-being.^17^

Another item that generated discussion was the item 41; the term “miserable” has a very strong connotation in the Portuguese language, and it was agreed among all the judges to replace it by “uncomfortable”, to smooth the question. 

The last step, the face-to-face meeting with the judges was essential to enrich the process, ensuring an improved pre-final version. The comprehension of all items was evaluated in the pre-test, and none was excluded. Only changes in the order of the sentences and/or search for synonym terms for a better understanding were done. 

It should be emphasized that the process of translation and cultural adaptation of an instrument requires a greater effort, involving more than just language and semantic aspects; it is necessary to adapt the terms from the cultural and conceptual point of view of the reality of the study population, considering its uniqueness to identify the construct to be measured.^18^

In this study, the validation of the QCG was carried out with nephropathic patients in hemodialysis therapy, which constituted a clientele subjected to long, chronic and difficult coping processes. The condition of assessing comfort level is essentially important in this context because people experience situations of stress, fear, ambivalence, malaise and anguish that are expressed in different levels depending on the particularities of individuals, and that can compromise their comfort state.^1^

Regarding the validity of the construct, the instrument obtained a satisfactory internal consistency index, presenting potential to be used in the practice of chronic renal patients, as well as in any other health context. In this cross-cultural adaptation, the *Cronbach’s* alpha was 0.80, higher than that found in the Turkish version of the Immobilization and Comfort Questionnaire, which was 0.75.^19^

The comfort construct has been applied to several clinical and epidemiologically distinct clienteles in order to verify how this dimension relates to care and self-care in the perspective of understanding how the level of comfort can positively or negatively influence the therapeutic processes in health.^20^

A systematic review identified that the relationships between self-care and the outcome comfort did not present strong evidence for a clientele that had undergone chemotherapy/radiation therapy to control cancer, thus raising the need for more comprehensive studies.^21^

Regarding the applicability of the Scale for Evaluation of Comfort in Patients Admitted to Psychiatric Clinic Services (ECIP), it was verified that the internal consistency of the instrument obtained a Cronbach’s alpha ranging from 0.72 to 0.91, and the psychospiritual and transcendence dimensions presented greater discomfort, that is, lower comfort.^22^

In turn, the *End of Life Comfort Questionnaire* in its Brazilian version was validated by means of the Kendall coefficient of agreement, with values ​​above the average in most of the items, which guarantees its application with safety to evaluate the construct comfort.^7^

More recently, the Comfort Scale for Relatives of People in Critical Health Status (ECONF), validated in Brazil with Cronbach’s alpha of 0.923, also showed high internal consistency and reliability, which allows its use to promote and evaluate the comfort offered by the interdisciplinary care team in the context of critical health situations.^23^

We considered, based on this research, that Comfort, as a nursing outcome, is applicable in different contexts (physical, socio-cultural, psycho-spiritual and environmental), and its effectiveness must be measured by means of validated instruments that allow to ensure the effectiveness of interventions related to it. 

It should be emphasized that the quality of the adaptation process determines the validity of the instrument to measure the construct in question. Thus, it is important that the instrument chosen to cultural adaptation has been well developed and comprehensively validated with satisfactory psychometric properties. It is important to consider the mentor’s comments on the instrument at each step of the process and discuss the conceptual significance of each item in the adaptation process. The author of the *GCQ* participated in this process and approved all the changes made in the Portuguese version.

This study has some limitations. The sample in which the psychometric test was conducted was restricted to patients from a single hemodialysis clinic, which limits the generalization of the results. Additional studies are needed to check the psychometric properties of the General Comfort Questionnaire - *Brazilian version* in other populations of renal patients and other clinical scenarios, aiming at the generalization of the scale. Another limitation to be mentioned is that the study did not consist in the allocation of items in the dimensions of the construct proposed by the comfort theory. This step, as well as the clinical validation will be, therefore, carried out in later studies. 

## Conclusion

The adaptation steps of the *GCQ* allowed the cultural adaptation to Brazilian reality with chronic kidney patients in hemodialysis therapy with a general equivalence of 94.3%. The judges evaluated the items contained in the questionnaire as pertinent, and ensured their semantic, cultural, idiomatic and conceptual coherence. After completion of all the steps, the instrument was named as follows: General Comfort Questionnaire - *Brazilian version*.

The General Comfort Questionnaire - *Brazilian version* revealed excellent level of comprehension and the items were considered relevant for clinical nursing practice. Thus, the General Comfort Questionnaire - *Brazilian version* is considered an instrument valid, reliable, reproducible, comprehensible and easy to apply to the Brazilian reality (α = 0.80). 

The validation of the Portuguese version of the instrument is presented as one additional resource to be made available to nurses acting in nephrology or in critical, clinical or surgical care; the instrument will help directing the decision-making to the nursing interventions to be performed according to the level of comfort and domains, either physical, socio-cultural, environmental and psycho-spiritual.
